# Effects of two neuromuscular training programs on running biomechanics with load carriage: a study protocol for a randomised controlled trial

**DOI:** 10.1186/s12891-016-1271-9

**Published:** 2016-10-22

**Authors:** Bernard X. W. Liew, Susan Morris, Justin W. L. Keogh, Brendyn Appleby, Kevin Netto

**Affiliations:** 1 School of Physiotherapy and Exercise Sciences, Curtin University, GPO Box U1987, Perth, WA 6845 Australia; 2 Faculty of Health Sciences and Medicine, Bond University, QLD 4229 Robina, Australia; 3 Sports Performance Research Centre New Zealand, AUT University, Auckland, New Zealand; 4 Cluster for Health Improvement, Faculty of Science, Health, Education and Engineering, University of the Sunshine Coast, Sippy Downs, Australia; 5 Strength and Conditioning, Australian Institute of Sport, Canberra, Australia; 6 High Performance Unit, Hockey Australia, Perth, Australia

**Keywords:** Running biomechanics, Load carriage, Randomized clinical trial, Neuromuscular training, Resistance training

## Abstract

**Background:**

In recent years, athletes have ventured into ultra-endurance and adventure racing events, which tests their ability to race, navigate, and survive. These events often require race participants to carry some form of load, to bear equipment for navigation and survival purposes. Previous studies have reported specific alterations in biomechanics when running with load which potentially influence running performance and injury risk. We hypothesize that a biomechanically informed neuromuscular training program would optimize running mechanics during load carriage to a greater extent than a generic strength training program.

**Methods:**

This will be a two group, parallel randomized controlled trial design, with single assessor blinding. Thirty healthy runners will be recruited to participate in a six weeks neuromuscular training program. Participants will be randomized into either a generic training group, or a biomechanically informed training group. Primary outcomes include self-determined running velocity with a 20 % body weight load, jump power, hopping leg stiffness, knee extensor and triceps-surae strength. Secondary outcomes include running kinetics and kinematics. Assessments will occur at baseline and post-training.

**Discussion:**

To our knowledge, no training programs are available that specifically targets a runner’s ability to carry load while running. This will provide sport scientists and coaches with a foundation to base their exercise prescription on.

**Trial registration:**

ANZCTR (ACTRN12616000023459) (14 Jan 2016)

**Electronic supplementary material:**

The online version of this article (doi:10.1186/s12891-016-1271-9) contains supplementary material, which is available to authorized users.

## Background

Ever since the “Battle of Marathon” between Greece and Persia was recorded [1], the accomplishment of running a 42 km marathon is seen as the ultimate achievement for a distance runner. However, the last two decades has seen both recreational and elite level runners striving for distances well beyond a standard marathon. Interest and participation in ultra-endurance races [2], multi-stage racing events [3], and off-terrain trail and adventure races have risen [4] as individuals seek new ways to challenge human limits. These races not only test a runner’s speed and endurance, but also their ability to navigate and survive over undulating terrains and harsh environments [3]. Navigation and survival requires routine access to specialized equipment and sustenance. This requirement necessitates athletes to compete with externally carried loads [5]. Few studies have considered the role of load carriage on the potential impact on a runner’s health and performance [6]. In addition, no studies have considered if runners can be trained to adapt to external loads in running.

Load carriage in running poses two fundamental problems to athletes and occupational personnel: 1) an increased injury rate, 2) and increased metabolic energy expenditure that may reduce performance [7]. In the adult population, the increased overuse injury rate associated with load carriage has largely been investigated in the military setting, where load carriage biomechanics have been predominantly investigated while walking [7]. A previous study reported that 8 % of the 5000 injuries reported in the Australian Defence Force from January 2009 to December 2010, were related to heavy load carriage [8]. Of these injuries, 56 % affected the lower limb and were classified as muscular stress related [8]. Although no causative studies have been performed, it is likely that load carriage while running may exacerbate the already high incidence of running related injuries [9]. In addition, when an individual runs with load, the energy demand involved in maintaining constant running speed is increased [10]. Minimising the reduction in running speed associated with load carriage is important for the survivability of military personnel, the performance of athletes, and the overall efficiency of movement in recreational runners [11, 12].

The risk of injury and reduced performance associated with load carriage in running, points to the need for a preconditioning program for these athletes. There is convincing evidence that resistance based neuromuscular training programs are effective at reducing running related injuries (RRI) during body weight (BW) running (i.e. running with no external load) [13], and improving BW running performance [14]. However, current training programs have been developed using BW running research [15, 16], rather than loaded running research. The only studies that have attempted to define best training practices for load carriage gait has been performed in the military setting [17]. A limitation in existing training studies has been that exercise prescription has not been explicitly informed from biomechanical studies of load carriage gait. Rather, training was of a generalised nature, targeting the large muscle groups of the lower limb [17]. The type of exercises and mode of contractions used for preconditioning programs should be specific to the gait pattern required for athletes, and be based on prior knowledge of biomechanical adaptations during load carriage.

### Potential adaptive and mal-adaptive biomechanics

Studies using computed muscle control and induced acceleration analysis have identified the integrated roles of lower limb muscles in BW running. Collectively, the functions of these muscles are to provide a vertical force to accelerate/decelerate body weight, and horizontal forces to accelerate/decelerate inertial mass [18, 19]. When additional load is imposed on a runner, greater vertical and horizontal forces are needed to accelerate and decelerate an increased total weight and total inertial mass, respectively. Biomechanical changes to running with load are classified as adaptive if they enable an increase in baseline motor function (Table 1). For example, an increase in ankle power absorption in mid stance with load may be adaptive as it transfers power away from proximal segments to the foot [6]. This may aid in increased elastic-energy recovery at the ankle plantar flexor muscle-tendon unit, which may be essential to sustain faster running velocities during load carriage.

**Table 1:** Biomechanical adaptations of load carriage to potentially optimize metabolic cost and minimise injury risk

Potential positive adaptation	Biomechanical changes with load	Potential negative adaptation
• Transfer energy from proximal to foot segment [64] • ↑ Energy stored as elastic energy [65]	↑ Ankle negative power mid-stance [6]	
• Accelerates leg into extension to ↑ energy transferred to proximal segments [64]	↑ Knee positive power late stance [6]	
• ↑ Hip extension deceleration of trailing thigh segment for preparation into hip flexion swing [66] • Transfers energy from trunk to trailing stance limb to prepare into swing [64]	↑ Hip negative power late stance [6]	
• ↑ Elastic energy recovery [67] • Avoid excessive vertical COM excursion and maintain ground reaction force alignment to stance limb [68, 69]	↑ Leg stiffness [70]	
• Architecture of triceps-surae muscle tendon unit makes it an efficient force generator [65]	Small role for inter-joint work redistribution [71]	
	↑ Hip adduction late stance [6]	• Asymmetrical loading on knee soft tissues [72]
	↑ Knee and ankle flexion mid-stance [6]	• ↑ COM vertical excursion [70] • ↑ Patellofemoral joint compression pressure and ↑ Achilles tendon compression [73, 74]

↑ = Increase; ↓ = Decrease

On the contrary not all biomechanical changes with load may be adaptive. Some mechanical changes are likely to be mal-adaptive as they may contribute to a greater risk of incurring RRI or represent an inefficient running style. Poor hip control of non-sagittal plane rotations has been documented to increase the risk of developing RRIs [20]. At the kinematic level, load carriage has been associated with increased hip adduction at terminal stance [6] (Table 1). This increase in non-sagittal plane movements may represent suboptimal muscle capacity and motor control [21]. In addition, poor proximal trunk-pelvis control in running may result in energy being wasted in maintaining postural balance and inter-segmental alignment. Remediating mal-adaptive mechanical changes whilst enhancing adaptive changes could improve biomechanical indices of running performance and injury risk during load carriage.

### Rationale

Load carriage in running is increasingly common in running related sports. The ability to positively and predictably adapt to the imposed load when running necessitates an evidence-based training program. Existing training studies for load carriage performance in the military setting cannot be immediately applied to load carriage running, as most studies investigated performance in walking. This is because running and walking involve different movement dynamics, making the extrapolation of results from walking studies problematic when applied to running. For example, the hip contributes approximately 20 % of total positive power in the stance phase of BW walking, but less than 10 % of total positive power in the same phase of BW running [22]. Second, studies that have investigated ways to improve load carriage performance have adopted a non-randomized design [17]. Reported effect sizes of benefit in intervention studies were larger in trials without a randomized design compared to one with a randomized design [23]. Lastly, studies on load carriage do not appear to specifically target the known neuromuscular demands of load carriage gait patterns [17]. Therefore, the purpose of this investigation is to compare the effects of a biomechanically informed neuromuscular training program to a generic standard best-practice resistance training program on changes in the biomechanics of running with load.

### Objectives

To compare changes in (1) self-determined running velocity with and without load carriage, (2) lower limb running kinematics and kinetics, (3) jumping power and hopping stiffness, (4) and isokinetic knee and ankle extensor strength in healthy adult runners participating in a biomechanically informed training program compared to a generic resistance training program. This generic resistance training program may be seen as the current “gold-standard” program based on current best evidence [17].

## Methods/design

### Research design

**Fig. 1:**
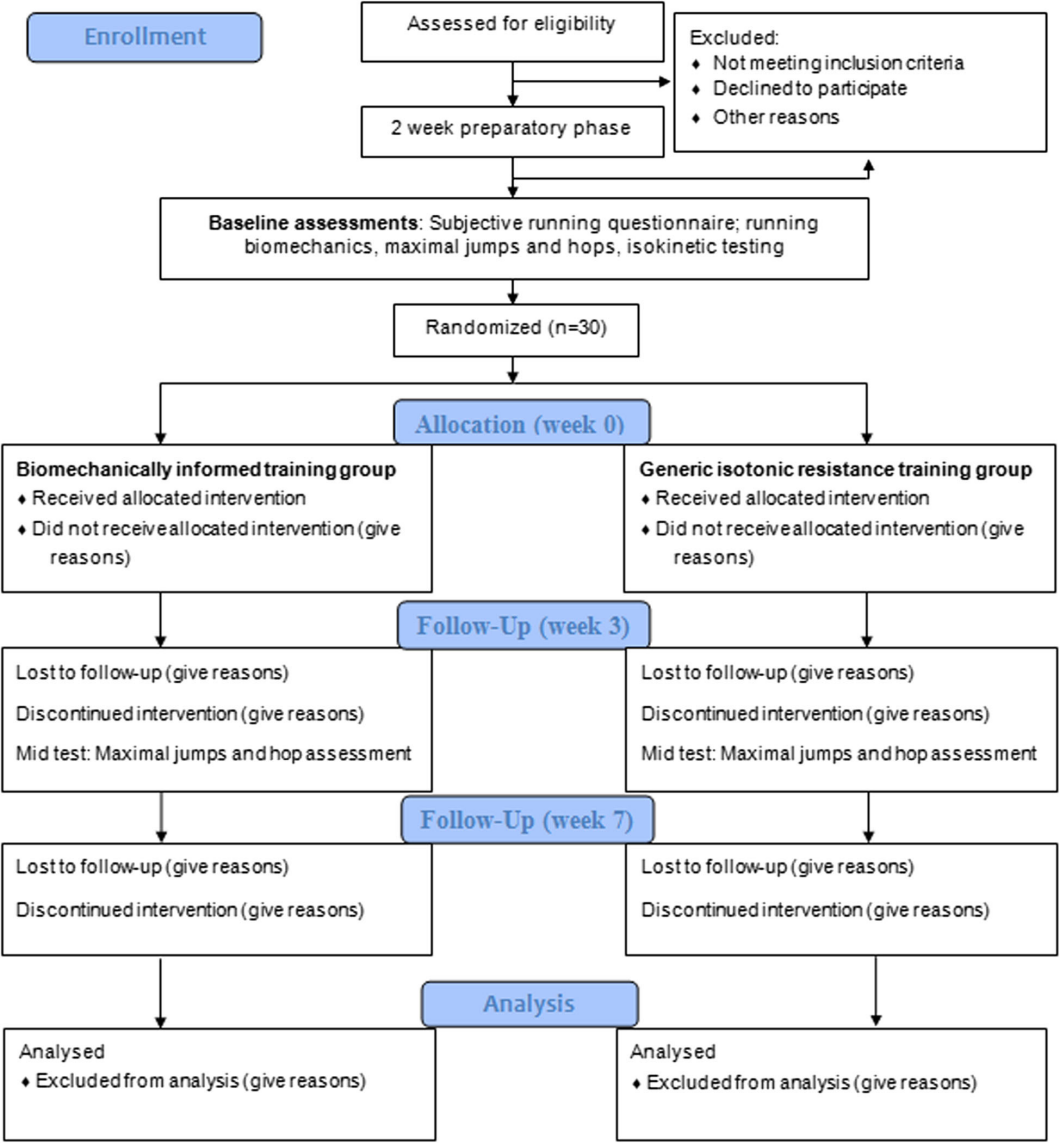
CONSORT Flow Diagram

The study is a single blinded, parallel-grouped randomized controlled trial which will be designed and reported according to the Consolidated Standards of Reporting Trials (CONSORT) statement (Fig. 1) [24]. This study is registered with the Australian New Zealand Clinical Trials Registry (ACTRN: ACTRN12616000023459). The Curtin University Human Research Ethics Committee (RD-41-14) has approved this study protocol. All participants will provide written informed consent prior to study inclusion.

### Participants, setting and recruitment

Runners with a variety of training experience residing in Western Australia will be invited to participate. All assessments and intervention will be conducted within Curtin University, Perth, Australia. Participants between 18 and 60 years old who are in good general health, and have been running or participating in running-related sports with a minimum cumulated total of 4 km/week or 45 min/week over the past 12 months, will be recruited. Exclusion criteria include: the presence of any disorders that could affect their gait and load carrying ability; medical conditions that preclude heavy resistance training and strenuous running; presence of a training-loss running related injury within the last three months [25]; current running related pain (except blisters or muscle soreness) [25]; lower limb surgery within the past 12 months; and females who are pregnant.

### Sample size calculation

This study was powered on the effects of a core stability program on changes to hopping leg stiffness [26]. Sample size was planned based on a two way, repeated measures ANOVA, using the Hotelling-Lawley Trace to test for an intervention by time interaction [27]. Previous studies on leg stiffness reported a standard deviation of 3600 N/m [26], and a correlation between repeated measures of 0.80 [28]. For a desired power of 0.80, and a Type 1 error rate of 0.05, 24 participants are needed to detect a between group mean difference of 3000 N/m. In order to account for a 20 % dropout over the six week intervention period, 30 participants will be tested.

### Randomization, allocation and blinding

Prior to randomization, participants will be stratified into two groups based on their gender. Previous studies have identified gender differences in BW running mechanics and different associative relationships between running mechanics and economy [16, 29]. Permuted block randomization will be performed within each stratum, using two different block sizes (two blocks of four and three blocks of eight) [30]. Each block consists of either four or eight group assignments (half of the assignments to one of two groups), to ensure that at the end of each block, the number of participants allocated to either intervention group will be balanced. The sequence within each block, and the order of all five blocks per stratum are randomized. The randomization sequence will be generated with an online random sequence generator used in previous a study [31]. When a participant has provided signed-informed consent, an external allocator not involved in the experiment, will sequentially draw an envelope (lowest numbered to highest) from either of two containers, depending on the stratum. The allocator will write the participant’s name, identifier number, date, and allocator’s signature on the envelope, which will be ink-printed onto the treatment allocation card via carbon paper. The envelope’s seal will be broken and the participant and the trainer will be informed about the allocated intervention group. Participant and trainer blinding will not be feasible due to the nature of prescribed intervention. Outcome assessor (for biomechanical and strength assessments) will be blinded to the allocation of participants to intervention groups.

### Subjective assessment

The following data will be collected at baseline: 1) participant’s demographics, 2) self-reported running training, load carriage, and strength training history; 3) self-reported medical status (including the Physical Activity Readiness Questionnaire); 4) self-reported running overuse injury history [25, 32].

### Three dimensional motion capture - load carriage running protocol

Participants will be carrying a backpack (CAMELBAK, H.A.W.G.® NV,14 l) fitted with chest strap and hip belt secured snuggly. Participants will be wearing their personal running attire and running shoes for all assessments. Participants will run over ground, in a straight line at two velocities: 1) self-determined velocity, and 2) 3.5 m/s, over two external load conditions (0 and 20 % BW). A lead up distance of at least 20 m to the edge of the first force plate and tail off distance of 10 m after the edge of the last force plate will be given to enable sufficient distance for acceleration and deceleration. A velocity of 3.5 m/s was predetermined as it closely represented the running velocity of the fastest men in 24 h ultra-marathons [33]. Sand bags will be filled to 20 % BW and secured within the backpack. A load of 20 % BW will be used as this represents a common relative load borne by tactical athletes during periods of running [11]. Indirect evidence from recommended backpack volume during ultra-endurance and adventure racers, suggest that a 20 % BW load is a reasonable approximate to actual load magnitude carried [5]. The order of load-velocity testing will be completely randomized using an online random sequence generator [6]. Timing gates (SMARTSPEED Pro, Fusion Sport Pty Ltd, Australia) will be positioned on both sides of the force plates (AMTI, Watertown, MA) (2000 Hz) 5 m apart, to monitor running velocity. Familiarisation trials will be given to practice the required running velocity. Participants will be required to complete a minimum of five successful running trials for each condition. Each trial would be interspersed with a 30 s rest break. A successful trial is defined when the right limb contacts the middle of the force platform without alteration of running pattern, within ±10 % of the prescribed speed. A minimum of five minutes rest will be given after each condition.

### Three dimensional motion capture – jumping and hopping protocol

Squat jumps (SJ), countermovement jumps (CMJ), and single leg vertical hoping (SL hop) will be performed on the force plates. For both the SJ and CMJ, the assessor will demonstrate and instruct technique, and participants will have to practise these jump techniques until performance is stable. For all tests, participants will be required to fix their arms at 90° abduction (“T” pose), to limit the influence of upper body on jump performance [34]. For the SJ, participants will perform a maximal concentric vertical jump from an initial squat depth of approximately 90° knee angle (visual estimation). For the CMJ, participants will descend into a squat depth of approximately 90° knee angle (visual estimation), followed without pause by a rapid maximal vertical jump and landing in a comfortable squat position [35]. When the assessor is satisfied with the practice performances, three maximal SJ trials and three maximal CMJ trials will be performed and recorded. Both SJ and CMJ will be performed with and without an external 20 % BW load. Each trial will be interspersed with a 30s rest and each test interspersed with a one minute rest to avoid fatigue. SL hopping will be performed for both legs separately over four conditions: 1) self-paced hopping frequency (BW), 2) self-paced hopping frequency with 20 % BW carriage, 3) hopping at a frequency of 3.0 Hz (BW), and 4) hopping at a frequency of 3.0 Hz with 20 % BW. Hopping frequency will be set using a handheld digital metronome. Each hop condition will last 10 s, with a one minute break interspersed [36]. Trials will be repeated if hopping frequency is not maintained. The order of testing will be randomized using an online random sequence generator (https://www.randomizer.org/).

### Data measurement and processing

An 18 camera motion capture system (Vicon T-series, Oxford Metrics, UK) (250Hz), synchronised to three consecutive in-ground force plates (three metre long in direction of progression) will be used to collect motion and force data for running, hopping and maximal jump tasks. Data will be captured and stored using manufacturer supplied software (Vicon Nexus, v2.3, Oxford Metrics, UK). Data processing will be performed in Vicon Nexus and Visual 3D (C-motion, Germantown, MD). Marker trajectories and ground reaction forces will be filtered at identical cut-off values, for use in inverse-dynamics calculations [37]. For joint angles, raw marker trajectories will be filtered at a separate cut-off frequency. The choice of cut-off frequencies for motion and force data will be based on past research [37]. A Cardan XYZ rotation sequence will be used to calculate 3D joint angles [38], and both kinematics and kinetics will be expressed in an orthogonal frame in the proximal segment [39]. Data will be computed during the stance and swing period of running. A threshold of 20 N in ground reaction force will used to determine initial contact and toe-off. Kinetic variables will be normalized using base factors of gravitational constant *g* (9.81 m/s^2^), leg length, *L* (m), and body mass, *M* (kg) [40].

### Biomechanical modelling

Individual retro-reflective markers will be attached to anatomical bony landmarks, and marker cluster-shells to limb segments of the thorax, pelvis, bilateral thigh, shank, and foot segments [18]. An eight segment, 27 degrees of freedom (DOFs) model will be constructed from a static standing trial [18]. The position and orientation of each segment will be calculated using an inverse kinematic (IK) algorithm in Visual 3D [41].

### Strength assessment (Isokinetic dynamometry)

An isokinetic dynamometer (HUMAC NORM, Computer Sports Medicine Inc., Stoughton, MA) will be used for strength testing, according to manufacturer’s guidelines. For the ankle plantar flexor assessment, participants will lay supine (inclination at 30° above horizontal) with the hip and knee positioned at 60° and 80° of flexion, respectively [42]. Based on the manufacturer’s manual, the dynamometer’s axis will be positioned in line with the lateral malleolus of the test limb. Next, the input arm penetration depth and foot plate penetration depth will be adjusted to approximate the dynamometer’s axis to the ankle axis visually. The testing ankle range will be set from 10° dorsiflexion to 30° plantarflexion. For knee extension testing, participants will be seated in the machine with the hip flexed to 85°. The axis of rotation will be aligned to the femoral condyles with the knee flexed at 90°. Testing range will be set from 0° (complete knee extension) to 90° knee flexion. For all testing, appropriate stabilization of segments will be applied using Velcro straps according to manufacturer’s testing guidelines, and gravity correction mode will be used [43]. Participants will first perform a standardised warm up protocol, consisting of 10 knee extension/flexion repetitions and ankle plantar flexion/dorsiflexion repetitions (90°/s), at a submaximal effort [44]. Concentric knee extensor and ankle plantar flexor torque will be assessed at an angular velocity of 60°/s. For each muscle assessment, two sets of six maximal concentric contractions will be performed, with a between set rest period of 1 min. Between test and between side rest periods of 3 min will be provided.

### Intervention

#### Familiarization phase (all participants) (two weeks)

All participants will first be enrolled into a two week preparatory training phase prior to baseline testing and randomization. During this phase, all participants will perform the same set of exercises (see Additional file 1 ‘Familiarization phase’). This preparatory phase will control for the effect of motor learning on improvements in performance on the assessments [45–47]. Participants will be encouraged not to alter any of their personal training regimes throughout the entire program. Self-reported training for the period of the intervention will be recorded by the participants in a supplemented training diary log book. Variables to report for self-resistance training include external mass magnitude, sets, and repetitions. Variables to report for cardiovascular training include duration, and type of exercise undertaken.

#### Training phase (six weeks) - standardized warm up (both groups)

Participants from both groups will begin each exercise session with a 15 min warm up consisting of four active, dynamic stretches consisting of 1) lunge, 2) ‘Good Morning’ hamstrings, 3) squats, and 4) calf raises off a step (see Additional file 1 ‘warm up’). Each dynamic stretch will be performed using only the BW as resistance. Each dynamic stretch will involve two sets of ten repetitions [48].

#### Generic neuromuscular resistance training group (GT group)

The principle governing this training program is that participants perform progressively heavier isoinertial (constant external mass) resistance training, at intensities from approximately 80 % progressing to approximately 88 % of one repetition maximum (1 RM). This program will involve three training sessions per week for six weeks (total 18 sessions). Inter-set rest duration of up to three minutes will be provided [49]. Exercises will include isoinertial bilateral leg press (Cybex® Plate Loaded Squat Press, Cybex International, Inc.), unilateral calf raises (Cybex® Plate Loaded Seated Calf, Cybex International, Inc.), and lunge (Cybex® Plate Loaded Smith Press, Cybex International, Inc.). For the leg press, foot placement will be shoulder width, and the depth of foot placement on the plate will be such that at 90° of knee flexion, the tip of the toes are in line with the knee and shoulders. For the calf raises, the foot will be positioned at the level of the 1^st^ metatarsophalangeal head (i.e. “ball” of the foot). For the lunge, the length of foot placements will be determined as a position that would enable approximately 90° knee flexion of the lead leg at the lowest position of the lunge. The foot of the trailing leg will be positioned such that the trailing knee is slightly posterior to the hip at the lowest position of the lunge. These exercises were selected as they represented generic lower limb exercises used in current load carriage training studies [17]. The intensity, repetitions, sets, rest duration, and description of each exercise will be gradually built up over the six weeks, and is described in the Additional file 1 (see ‘Exercises progression table (General training group)’).

#### Biomechanically informed neuromuscular resistance training group (BIT group)

The principle of this training program is that key neuromuscular requirements of load carriage running are targeted by specific neuromuscular exercises. This program will involve three training sessions per week for six weeks (total 18 sessions), and will involve SL hopping, CMJ, and hip flexor pull (Cybex® Bravo Pull, Cybex International, Inc.). For the plyometric component (hop and CMJ), intensity will be varied using a weighted vest. A maximal mass of 20 % BM will be added, as previous studies have demonstrated a reduction in peak power with heavier loads [50]. A previously published review indicated that sessions incorporating more than 50 foot contacts per session resulted in the most benefit for jump performance [51]. In order to maintain peak power application for the CMJ and hip flexor pull, a cluster set method (multiple sets of two to three repetitions with 10 s inter-set breaks) will be used [52]. The hip flexor pull will involve a range of 5° hip extension to 90° hip flexion (visual estimation), and a single hand-hold support will be used to maintain balance. For the CMJ, a depth of 80° to 90° knee flexion will be visually estimated and used for all participants. For SL hopping, participants will be encouraged to generate hopping power from the ankle joint, with the knee kept in a relatively ‘isometric’, slightly flexed posture. SL hopping will not involve a cluster-set method as the exercise intrinsically involves continuous, repetitive cycles of fast stretch-shortening cycle. The intensity, repetitions, sets, rest duration, and description of each exercise will be gradually built up over the six weeks, and is listed in the Additional file 1 (see ‘Exercises progression table (Biomechanically informed training group)’).

#### Augmented feedback (both groups)

Augmented feedback (AF) during all exercises for both groups will be provided to participants, using the principles of motor skill learning [53] (see Additional file). This is to enhance the learning and retention of optimal exercise performance in both groups, with the intention that sub-optimal lower limb kinematics with load may be corrected post-intervention. First, AF that directs an individual’s attention to the consequence of a movement (i.e. external focus of attention) has been shown to result in better motor learning and retention. Second, AF will be provided before (demonstration and instruction), during (mirror feedback and physical/verbal guidance), and after (knowledge of performance) each set of exercise in the initial stages, progressing to feedback delivered only after each exercise set. Feedback based on knowledge of performance (KP) will be provided in a prescriptive sense (i.e. what you should perform) at the initial stages, progressing to descriptive sense (i.e. what was performed) in the later stages of training. This is to allow participants to self-formulate correctional motor strategies in the later stages. The frequency of AF will be reduced from occurring at every set in the early stage of learning, to the last set of an exercise in the later stages. Previous research has shown that introducing a time delay from motor task completion to feedback delivery, especially when participants self-evaluate their performance during this time lag, improves motor skill learning [53].

#### Determining initial training loads and progression

During the familiarization phase, for all isoinertial exercises a 10 RM will be utilised. A 1 RM load will then be derived from a 10RM load using a regression table for novice strength trainers (Table 2) [54]. Load intensity will progress from 80 % of estimated 1 RM (equivalent to a 10RM load) in the first two weeks, to 84 % of estimated 1RM in the next two weeks (equivalent to an 8RM load), to 88 % of estimated one RM (equivalent to a 6RM load) in the final two weeks. The number of repetitions performed per set will be two repetitions less than the repetition maximum [55]. Estimated 1 RM load for each exercise will be adjusted by a weekly increase of approximately 2.5 % to account for progression in strength. The rate of progression was based on a previous study on time course for strength gains, which demonstrated approximately 20 % increase in measured 1 RM in six weeks [56]. For the hopping and CMJ, a weekly increase in load carried (approximately 5 % BW per week) will be used, until a limit of 20 % BW is reached.

**Table 2:** Guide for determining one repetition maximum in novice weight trainers [54]

% 1RM	100	96	94	92	90	88	86	84	82	80	78	76	74	72	70	68	66	64	62	61
Number Repetitions	1	2	3	4	5	6	7	8	9	10	11	12	13	14	15	16	17	18	19	20
Reconvert factor	-	1.04	1.06	1.08	1.11	1.13	1.16	1.19	1.22	1.25	1.28	1.31	1.35	1.39	1.43	1.47	1.52	1.56	1.61	1.63

### Between group differences in training volume

Training volume as quantified by the number of sets, repetitions, and load magnitude will not be exactly matched between groups. This could mediate any potential between group intervention effects [57]. However, the aim of this study is to test two ecologically realistic model of training on load carriage running mechanical outcomes.

### Intervention adherence

Participants attending ≥ 70 % of all training sessions (≥13/18 sessions) will be classified as high adherence, whereas those attending < 70 % will be classified as low adherence. Adherence will be calculated from attendance records in each participant’s exercise training records. Adherence to prescribed neuromuscular training has been previously reported to be an important effect modifier in these programs [58, 59]. Efforts to increase participant adherence include, weekly mobile text (short messaging service) reminders and an exercise diary.

### Dependent variables and statistics

For the SJ and CMJ, peak power using inverse dynamics and the force plate approach will be derived [60]. For SL hopping, leg stiffness at each condition will be derived. For the self-paced running tasks with and without load carriage, average self-paced running velocity will be derived over a complete stride. Discrete variables of individual joint positive and negative work, total and net joint work for stance and swing phase of all running trials will be derived. Spatio-temporal variables of stance and swing duration, stride length, and cadence will be derived for all running trials. Time series of the three dimensional joint angles, moments, and powers of all three joints will be extracted for all running trials. Three-dimensional leg stiffness in running will be calculated in a three-step process from an adapted method in a previous study [61]. First, a three dimensional leg length will be defined as the vector from the hip joint centre to the centre of pressure. Second, the component of the resultant three dimensional ground reaction force (GRF) projected onto the leg vector will be calculated (taking the dot product of the GRF vector with the unit vector of the leg). Lastly, leg stiffness will be derived using the ratio of projected GRF (at the time of peak resultant GRF) to the change in leg length (between initial contact to peak GRF). For strength analysis, average peak concentric torque and power, and absolute peak concentric torque and power, of the knee extensors and ankle plantar flexors will be extracted.

Descriptive statistics (mean and standard deviation) will be calculated for baseline demographics of participants. Between groups difference in baseline demographics will be calculated using *t*-test or non-parametric test where appropriate. Analysis will be based on an intention-to-treat (ITT) using the multiple imputation method [62]. A repeated measures linear mixed model with time, group, and their interaction as fixed effects, and participants clustered within groups as random effects will be used to analyse our discrete dependent variables [63]. For the linear mixed model, significance will be set at α = 0.05. Descriptive statistics and linear mixed modelling will be performed in R software within RStudio (Version 0.98.1062, RStudio, Inc.). Statistical testing between group and within group mechanical wave form data (kinematics, kinetics) will be analysed using Statistical Parametric Mapping (SPM). Statistical significance will be inferred using Random Field Theory (RFT), with appropriate Bonferroni correction applied to retain a family-wise error rate of α = 0.05. SPM will be performed using the latest version of spm1d package (www.spm1d.org), installed in Python 2.7, and implemented in Enthought Canopy 1.5.4 (Enthought Inc., Austin, USA).

## Discussion

Carrying some form of external load is becoming increasingly ubiquitous in running related sports, such as adventure racing and ultra-endurance events. Running mechanics alterations with load carriage could represent adaptive or mal-adaptive mechanics. Mechanical changes like increased joint power may represent attempts at maintaining constant running velocity, support an increased weight, maintain postural control and/or attenuate excessive impact shocks. In addition, some mechanical changes are likely to represent a failed capacity of lower limb muscles to cope with the additional load, that result in a reduction in running performance and an increased risk of future injuries. The long term sequela not only has an effect at an individual level, but could affect long term sporting participation and health care costs. In addition, runners may have to compromise running economy and running velocity when load carriage is involved if lower limb muscles are not tuned to the specific neuromuscular demands. Velocity decrements as a result of load carriage would result in compromised survivability in combat soldiers, and reduced performance in competing running athletes. This study will provide preliminary evidence of the potential efficacy of a targeted neuromuscular training program or a best-practice strength training program on improvements in strength, stiffness, running velocity and biomechanics during load carriage running.

## Additional file

Additional file 1: Effects of two neuromuscular training programs on running biomechanics with load carriage: a randomized controlled trial – a study protocol. (DOCX 312 kb)

## Abbreviations

AF: Augmented feedback
BW: Body weight
CMJ: Countermovement jumps
CONSORT: Consolidated Standards of reporting trials
GRF: Ground reaction force
KP: Knowledge of performance
RM: Repetition maximum
RRI: Running related injuries
SJ: Squat jumps
SL hop: Single leg vertical hoping
