# Supplementary material for: Effects of two neuromuscular training programs on running biomechanics with load carriage: a study protocol for a randomised controlled trial
Source: BMC Musculoskelet Disord. 2016 Oct 22;17:445. doi: 10.1186/s12891-016-1271-9 (PMC5075409; doi:10.1186/s12891-016-1271-9)
Supplement: Additional file 1: — Effects of two neuromuscular training programs on running biomechanics with load carriage: a randomized controlled trial – a study protocol. (DOCX 312 kb) [file 12891_2016_1271_MOESM1_ESM.docx]

**Supplementary material**

**Title:** Effects of two neuromuscular training programs on running biomechanics with load carriage: a randomized controlled trial – a study protocol.

**Summary of contents:** Tables and figures detailing the type, frequency, set, repetition, relative intensity and rest duration for participants in both experimental (Biomechanically informed training) and control (General training) groups.

**Authors:** Bernard X.W. Liew, BSc (Hon),^a^ Susan Morris, PhD,^a^ Justin W.L. Keogh, PhD,^b,c,^ Brendyn Appleby, MSc,^e,f^ Kevin Netto, PhD ^a^

**Affiliations:**

^a^ School of Physiotherapy and Exercise Sciences, Curtin University, GPO Box U1987, Perth, WA 6845, Australia.

^b^ Faculty of Health Sciences and Medicine, Bond University, QLD 4229, Australia

^c^ Sports Performance Research Centre New Zealand, AUT University, Auckland, New Zealand

^d^ Cluster for Health Improvement, Faculty of Science, Health, Education and Engineering, University of the Sunshine Coast

^e^ Strength and Conditioning, Australian Institute of Sport, Canberra, Australia

^f^ High Performance Unit, Hockey Australia, Perth, Australia

Address correspondence and reprint requests to Mr Bernard Liew, School of Physiotherapy and Exercise Sciences, Curtin University, GPO Box U1987, Perth, WA 6845, Australia; E-mail: b.liew@postgrad.curtin.edu.au Tel: +618 9266 3689; Fax: +618 9266 3699.

**Familiarization phase**

2 weeks (4 sessions)

| Session | SL hop (Body Weight) | CMJ (Body Weight) | Hip flexor pull | Leg press | Calf raise | Lunge |
| --- | --- | --- | --- | --- | --- | --- |
| 1.1 | 2 sets of 20 hops | 3 sets of 3reps | 1 set of 10 reps (15RM load)  1 set of 10 reps (15RM load) | 1 set of 10 reps (15RM load)  1 set of 10 reps (15RM load) | 1 set of 10 reps (15RM load)  1 set of 10 reps (15RM load) | 1 set of 10 reps (15RM load)  1 set of 10 reps (15RM load) |
| 1.2 | 2 sets of 20 hops | 3 sets of 3reps | 1 set of 10 reps (15RM load)  1 set of 10 reps (10RM load) | 1 set of 10 reps (15RM load)  1 set of 10 reps (10RM load) | 1 set of 10 reps (15RM load)  1 set of 10 reps (10RM load) | 1 set of 10 reps (15RM load)  1 set of 10 reps (10RM load) |
| 2.1 | 2 sets of 20 hops | 3 sets of 3reps | 1 set of 10 reps (15RM load)  1 set of 10 reps (10RM load) | 1 set of 10 reps (15RM load)  1 set of 10 reps (10RM load) | 1 set of 10 reps (15RM load)  1 set of 10 reps (10RM load) | 1 set of 10 reps (15RM load)  1 set of 10 reps (10RM load) |
| 2.2*** | 2 sets of 20 hops | 3 sets of 3reps | 1 set of 10 reps (10RM load) | 1 set of 10 reps (15RM load)  1sets of 10 reps (12RM load)  2sets of 10 reps (10RM load) | 1 set of 10 reps (10RM load) | 1 set of 10 reps (10RM load) |
| Note | Used as warm up and technique phase | Used as warm up and technique | Machine | Machine | Machine | Machine |
| SL = Single leg; CMJ = Countermovement jumps; RM = repetition maximum; reps = repetitions | | | | | | |

**Training phase**

Warm up

| **Lunge**  X 2 sets  10 repetitions per side | **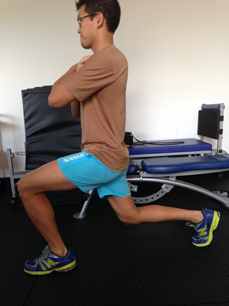** |
| --- | --- |
| **Good Morning**  X 2 sets  10 repetitions | 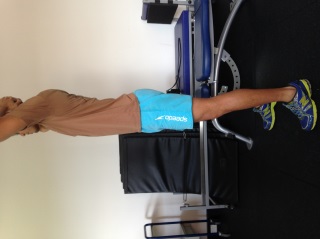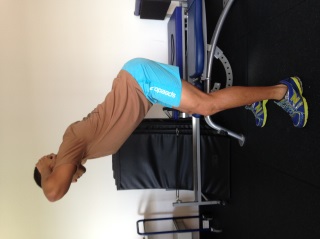 |
| **Squats**  X 2 sets  10 repetitions | 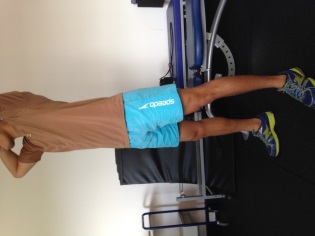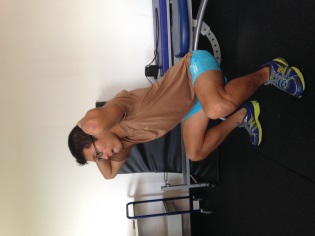 |
| **Bilateral calf raise**  X 2 sets  10 repetitions | 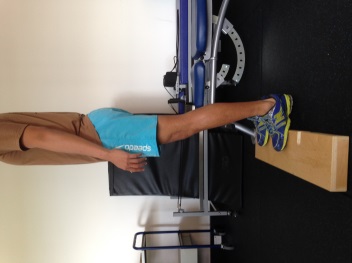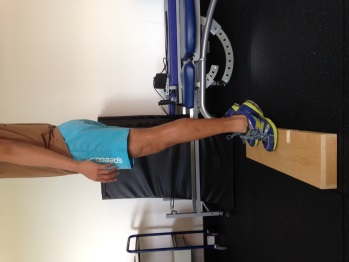 |

*Exercises progression table (Biomechanically informed training group)*

| Exercise/  Week (session) | SL hopping (Sets.Time.Frequency) | Total % BW performed for hops | CMJ  (Sets.Reps) | Total % BW performed for CMJ | Hip flexor pull  (Sets.Reps.Load) | Total reps per limb |
| --- | --- | --- | --- | --- | --- | --- |
| 1.1 | 2.20s.2.2Hz | 110 | 10.2 | 100 | 8.2.10RM | 124 |
| 1.2 | 2.20s.2.2Hz | 110 | 5.3 | 100 | 8.2.10RM | 119 |
| 1.3 | 2.20s.2.2Hz | 110 | 10.2 | 100 | 8.3.10RM | 132 |
| Total week 1 | **264** |  | **55** |  | **56** | **375** |
| 2.1 | 2.20s.2.2Hz | 110 | 10.2 | 105 | 10.2.10RM | 128 |
| 2.2 | 2.20s.2.2Hz | 110 | 5.3 | 105 | 10.2.10RM | 123 |
| 2.3 | 3.20s.2.2Hz | 110 | 10.2 | 105 | 10.3.10RM | 182 |
| Total week 2 | **308** |  | **55** |  | **70** | **433** |
| 3.1 | 2.20s.2.2Hz | 115 | 10.2 | 110 | 8.2.8RM | 124 |
| 3.2 | 2.20s.2.2Hz | 115 | 5.3 | 110 | 8.2.8RM | 119 |
| 3.3 | 4.20s.2.2Hz | 115 | 10.2 | 110 | 8.3.8RM | 220 |
| Total week 3 | **352** |  | **55** |  | **56** | **463** |
| 4.1 | 2.20s.2.2Hz | 120 | 10.2 | 115 | 10.2.8RM | 128 |
| 4.2 | 2.20s.2.2Hz | 120 | 5.3 | 115 | 10.2.8RM | 123 |
| 4.3 | 4.20s.2.2Hz | 120 | 10.2 | 115 | 10.3.8RM | 226 |
| Total week 4 | **352** |  | **55** |  | **70** | **477** |
| 5.1 | 2.20s.3Hz | 120 | 10.2 | 120 | 8.2.6RM | 156 |
| 5.2 | 2.20s.3Hz | 120 | 5.3 | 120 | 8.2.6RM | 151 |
| 5.3 | 4.20s.3Hz | 120 | 10.2 | 120 | 8.3.6RM | 284 |
| Total week 5 | **480** |  | **55** |  | **56** | **591** |
| 6.1 | 3.20s.3Hz | 120 | 10.3 | 120 | 10.2.6RM | 220 |
| 6.2 | 2.20s.3Hz | 120 | 10.2 | 120 | 10.2.6RM | 160 |
| 6.3 | 3.20s.3Hz | 120 | 10.2 | 120 | 10.3.6RM | 240 |
| Total week 6 | **480** |  | **70** |  | **70** | **620** |
| Total weeks 1-6 |  |  |  |  |  | **2959 reps** |
|  | Inter set rest = 3 mins  Inter exercise rest of 3 to 5 mins |  | Inter set rest = 10 s  Inter exercise rest of 3 to 5 mins |  | Inter set rest = 3 mins  Inter exercise rest of 3 to 5 mins |  |
| SL = Single leg; CMJ = Countermovement jumps; RM = repetition maximum; reps = repetitions; %BW = percentage body weight; mins = minutes | | | | | | |

*Exercises progression table (General training group)*

| Exercise/  Week (session) | Leg press  (Sets.Reps.Load) | Calf raise  (Sets.Reps.Load) | Lunge  (Sets.Reps.Load) | Total reps per limb |
| --- | --- | --- | --- | --- |
| 1.1 | 2.8.10RM | 2.8.10RM | 2.8.10RM | 48 |
| 1.2 | 2.8.10RM | 2.8.10RM | 2.8.10RM | 48 |
| 1.3 | 3.8.10RM | 2.8.10RM | 2.8.10RM | 56 |
| Total week 1 | **56** | **48** | **48** | **152** |
| 2.1 | 3.8.10RM | 2.8.10RM | 2.8.10RM | 56 |
| 2.2 | 2.8.10RM | 2.8.10RM | 2.8.10RM | 48 |
| 2.3 | 3.8.10RM | 3.8.10RM | 3.8.10RM | 72 |
| Total week 2 | **64** | **56** | **56** | **176** |
| 3.1 | 2.6.8RM | 3.6.8RM | 3.6.8RM | 48 |
| 3.2 | 2.6.8RM | 2.6.8RM | 3.6.8RM | 42 |
| 3.3*** | **3.10.10RM***** | 2.6.8RM | 1.6.8RM | 48 |
| Total week 3 | **54** | **42** | **42** | **138** |
| 4.1 | 2.6.8RM | 2.6.8RM | 2.6.8RM | 36 |
| 4.2 | 4.6.8RM | 3.6.8RM | 2.6.8RM | 54 |
| 4.3 | 4.6.8RM | 4.6.8RM | 3.6.8RM | 66 |
| Total week 4 | **60** | **54** | **42** | **156** |
| 5.1 | 3.4.6RM | 3.4.6RM | 3.4.6RM | 36 |
| 5.2 | 3.4.6RM | 2.4.6RM | 2.4.6RM | 28 |
| 5.3 | 4.4.6RM | 3.4.6RM | 3.4.6RM | 40 |
| Total week 5 | **40** | **32** | **32** | **104** |
| 6.1 | 4.4.6RM | 4.4.6RM | 4.4.6RM | 48 |
| 6.2 | 2.4.6RM | 2.4.6RM | 2.4.6RM | 24 |
| 6.3*** | **3.10.10RM***** | 3.4.6RM | 3.4.6RM | 54 |
| Total week 6 | **54** | **36** | **36** | **126** |
| Total |  |  |  | **852** |
|  | Inter set rest of 3 mins  Inter exercise rest of 3 to 5 mins | | | |
| RM = repetition maximum; reps = repetitions; %BW = percentage body weight; mins = minutes | | | | |

*Augmented feedback (AF) cues for biomechanically informed training group*

| **Exercises** | **Optimal technique** | **Feedback cues** |
| --- | --- | --- |
| **Leg press** | 1. Sit erect with back pressed against back of seat 2. Feet should width apart, hips slighted external rotated. Tip of toes, knee, and should be in a straight line at 90̊ knee flexion. 3. Push footplate away by extending the hips and knees. 4. Do not lock knees 5. Bring footplate back till 90̊ knee flexion, and smoothly transition back to full extension. | **Verbal:**   1. “Keep knees over toes” |
| **Calf raise** | 1. Set erect, and position foot such that 1^st^ MTP head is at the edge of the foot plate. 2. Push up from the balls of the feet to raise the heel as high as possible. 3. Avoid lifting thigh and leaning backwards when pushing up. | **Verbal:**  1. “Tighten foot arch as you push up”  2. “ Keep trunk tall” |
| **Lunge** | 1. Set anterior-posterior foot width such that 90̊ knee flexion of lead limb at lowest point of descent. 2. Lower hips down till forward limb’s thigh parallel to floor. 3. Flex trail limb’s knee, without touching floor. 4. Raise hips by pushing off with both limbs while exhaling to return to start position. 5. Maintain erect trunk. | **Verbal:**  1. “Trunk and hips stay tall”  2. “Keep knee over toes” |

*Augmented feedback (AF) cues for general training group*

| **Exercises** | **Optimal technique** | **Feedback cues** |
| --- | --- | --- |
| **Single leg hop** | 1. Bilateral shoulder and pelvis level in single leg stance 2. Hip, knee, and ankle joint centre should be in the same sagittal plane 3. Avoid excessive knee flexion | **Verbal:**   1. “Trunk and hips stay tall” 2. “Spring from your ankle”; “stay tall at the knees" 3. “Keep landing soft” 4. “Spring up as fast as you can“   **Visual:**   1. Use of mirror during exercise   **Auditory:**   1. Ensure soft landing |
| **Countermovement jump** | Preparatory and eccentric phase:   1. Feet approximately should width apart 2. Adequate trunk, hip, knee, ankle flexion on descent 3. Adequate shoulder extension   Amortization:   1. Transit from eccentric to concentric phase fast with no pause   Concentric phase:   1. Full trunk, hip, knee, ankle extension during flight 2. No lateral lean of trunk 3. Full shoulder flexion   Landing:   1. Soft landing 2. Landing position same as preparatory position 3. Should land as close as starting position with little AP and lateral translation | **Verbal:**  1. “Feet and knees shoulder width apart”;  2. “Maintain weight over centre of foot”;  3. “Keep landing soft”  4. “Spring up as fast as you can”, “drive with your shoulders”  5. “Stay tall in the air”  6. “ Avoid kissing the knees”  **Visual:**  1. Use of mirror during exercise  2. Check foot position and compare to original start position  **Auditory:**  1. Ensure soft landing  2. Ensure only one contact sound |
| **Hip flexor pull** | 1. Stand on one leg maintaining vertical trunk, level pelvis alignment 2. From a position of hip extension, flex the opposite hip and knee to 90 degree flexion 3. Return opposite limb to a position of hip extension 4. Maintain global alignment and postural control 5. Limit use of trunk flexion and lateral flexion during exercise | **Verbal:**  1. “Trunk and hips stay tall”;  2. “Pull up with your thigh”  3. “Grip the floor with your standing feet” |

Augmented feedback (AF) principle and schedule (Both groups)

| **Weeks** | **Focus of attention** | **Period of AF** | **Type of KP** | **Frequency of AF** | **Delay in AF** |
| --- | --- | --- | --- | --- | --- |
| **Familiarization** | External | Before movement (Observation and demonstration); During movement (visual, verbal, physical guidance); After movement (KP verbal) | Descriptive and Prescriptive | Every set of an exercise | Almost instantaneous |
| **1** | External | Before movement (Observation and demonstration); During movement (visual, verbal, physical guidance); After movement (KP verbal) | Prescriptive | Every alternate set of an exercise | Delay ~ 5 to 10 sec |
|  |  |  |  |  |  |
| **6** | External | After movement (KP verbal) | Descriptive | Last set of an exercise (summary feedback) | Delay + concurrent subjective performance estimations |
| KP: Knowledge of performance | | | | | |
